# Quantifying biases in marine‐protected‐area placement relative to abatable threats

**DOI:** 10.1111/cobi.13340

**Published:** 2019-05-27

**Authors:** Caitlin D. Kuempel, Kendall R. Jones, James E.M. Watson, Hugh P. Possingham

**Affiliations:** ^1^ Centre for Biodiversity and Conservation Science School of Biological Sciences University of Queensland St. Lucia QLD 4072 Australia; ^2^ ARC Centre of Excellence for Environmental Decisions University of Queensland St. Lucia QLD 4072 Australia; ^3^ National Center for Ecological Analysis and Synthesis University of California 735 State Street, Suite 300 Santa Barbara CA 93101 U.S.A.; ^4^ School of Earth and Environmental Sciences, Steele Building University of Queensland Brisbane Queensland 4072 Australia; ^5^ Global Conservation Program Wildlife Conservation Society 2300 Southern Boulevard Bronx NY U.S.A.; ^6^ The Nature Conservancy South Brisbane Queensland 4101 Australia

**Keywords:** Aichi Target 11, conservation impact, conservation planning, conservation targets, convention on biological diversity, counterfactual, protected area effectiveness, contrafactual, Convenio sobre la Diversidad Biológica, efectividad del área protegida, impacto de conservación, Objetivo 11 de Aichi, objetivos de conservación, planeación de la conservación, 爱知目标 11, 保护规划, 保护成效, 保护目标, 生物多样性公约, 违实分析, 保护区有效性

## Abstract

Marine protected areas (MPAs) are a critical defense against biodiversity loss in the world's oceans, but to realize near‐term conservation benefits, they must be established where major threats to biodiversity occur and can be mitigated. We quantified the degree to which MPA establishment has targeted stoppable threats (i.e., threats that can be abated through effectively managed MPAs alone) by combining spatially explicit marine biodiversity threat data in 2008 and 2013 and information on the location and potential of MPAs to halt threats. We calculated an impact metric to determine whether countries are protecting proportionally more high‐ or low‐threat ecoregions and compared observed values with random protected‐area allocation. We found that protection covered <2% of ecoregions in national waters with high levels of abatable threat in 2013, which is ∼59% less protection in high‐threat areas than if MPAs had been placed randomly. Relatively low‐threat ecoregions had 6.3 times more strict protection (International Union for Conservation of Nature categories I–II) than high‐threat ecoregions. Thirty‐one ecoregions had high levels of stoppable threat but very low protection, which presents opportunities for MPAs to yield more significant near‐term conservation benefits. The extent of the global MPA estate has increased, but the establishment of MPAs where they can reduce threats that are driving biodiversity loss is now urgently needed.

## Abstract (es)

Sesgos de Cuantificación en la Ubicación de Áreas Marinas Protegidas en Relación con las Amenazas Abatibles a la Biodiversidad

**Resumen**

Las áreas marinas protegidas (MPAs, en inglés) son un sistema de defensa crítica contra la pérdida de biodiversidad en los océanos del mundo, pero para hacer realidad los beneficios de conservación de corto plazo, estas áreas deben establecerse en donde ocurren la mayoría de las amenazas para la biodiversidad y en donde puedan mitigarse. Cuantificamos el grado al cual la fundación de MPAs se ha enfocado en amenazas abatibles (es decir, amenazas que pueden abatirse solamente por medio de MPAs manejadas efectivamente) al combinar los datos de amenazas para la biodiversidad marina espacialmente explícita en 2008 y en 2013 y la información sobre la ubicación y el potencial que tienen las MPAs para detener las amenazas. Calculamos una medida de impacto para determinar si los países están protegiendo proporcionalmente más ecoregiones de alta o baja amenaza y comparamos los valores observados con las áreas protegidas asignadas al azar. Descubrimos que la protección cubría <2% de las ecoregiones en aguas nacionales con niveles altos de amenazas abatibles en 2013, lo cual es ∼59% menos protección en las áreas de alta amenaza que si las MPAs se hubieran ubicado al azar. Las ecoregiones con una baja amenaza relativa tuvieron 6.3 veces más protección estricta (categorías I‐II de la Unión Internacional para la Conservación de la Naturaleza) que las ecoregiones con amenaza alta. Treinta y un ecoregiones tuvieron niveles altos de amenazas abatibles pero muy baja protección, lo cual representa oportunidades para que las MPAs tengan más beneficios significativos a corto plazo. La extensión del conjunto global de MPAs ha incrementado, pero la fundación de MPAs en lugares donde pueden reducir laamenazas que causan la pérdida de biodiversidad es una necesidad urgente en día.

## 摘要

海洋保护区建立是防止地球海洋生物多样性丧失的一项重要措施， 但要达到短期的保护成效， 必须在生物多样性面临严重威胁且威胁可被减轻的地区建立保护区。结合 2008 年和 2013 年海洋生物多样性威胁的空间数据及海洋保护区的位置和减缓威胁的潜力等信息， 我们量化分析了海洋保护区的建立对于减缓威胁的效果。我们用一个影响指标来计算各个国家保护的， 受高水平和低水平威胁的生态区比例大小， 并将观测值与随机设置保护区的情况进行比较。我们发现， 2013 年, 可控威胁水平较高的国家海域中仅有低于 <2% 的生态区得到保护， 这比随机设置海洋保护区所保护的受高威胁地区少了约 59% 。受相对低威胁的生态区比受高威胁生态区得到的严格保护 （世界自然保护联盟 I‐II 类标准） 高 6.3 倍。有三十一个生态区存在高水平的可控威胁， 但得到保护却很少， 这为海洋保护区在短期内取得更显著的保护成效带来了机会。目前， 全球海洋保护区的范围正在扩大， 但仍迫切需要在导致生物多样性下降的， 威胁可以得到有效减缓的地区建立保护区。**【翻译: 胡怡思; 审校: 聂永刚】**

## Abstract (es)

*Article impact statement*: Strategic guidelines and better evaluation metrics are needed to correct biases in marine protection relative to levels of stoppable threat.

## Introduction

Marine protected areas (MPAs) are a cornerstone of conservation and now cover >7% of the world's oceans (UNEP‐WCMC 2018). Several countries have recently received global attention for declaring vast MPAs. For example, the United States expanded the Pacific Remote Islands Marine National Monument to cover >1 million km^2^ and 80% of Palau's Exclusive Economic Zone (EEZ) was designated a no‐take area (UNEP‐WCMC & IUCN 2016). Despite this growth, MPA coverage remains below the United Nation's Strategic Plan for Biodiversity's Aichi Target 11, which mandates protection of at least 10% of marine area by 2020 (Convention on Biological Diversity 2010).

The conservation effectiveness of PAs is often questioned because expansion frequently targets areas that are unlikely to be affected in the short or medium term; hence, they deliver little conservation benefit relative to no action (e.g., Pressey et al. 2002; Ferraro & Pattanayak 2006; Barnes 2015). Approaches, such as National Geographic's Pristine Seas program, which targets areas where political costs of large PAs are low and threats are anticipated to expand, will likely have significant long‐term benefits (Sacre et al. 2019). However, such efforts have also been criticized for potentially redirecting limited conservation resources from areas under immediate threat that are in great need of protection and for producing low return on conservation investments in the near term (Pressey 1994; Devillers et al. 2015). To realize a near‐term net conservation benefit PAs need to be established where conservation value is expected to decrease in the absence of action (i.e., area is under threat) and where conservation action (e.g., PA establishment) can reduce threats (Maron et al. 2013).

Protected areas have a range of management objectives, from strict biodiversity conservation (IUCN categories I–II) to zones that allow some levels of sustainable use (IUCN categories III–VI). However, the primary objective of all PAs with an IUCN category is to conserve nature (Dudley 2008; Jones et al. 2018b), and the overarching goal of the Convention on Biological Diversity's Aichi target 11, a major driver of PA establishment, is to “safeguard ecosystems, species, and genetic diversity” and “improve the status of biodiversity” (CBD Secretariat 2010). Similarly, ecosystem‐based management approaches aim to reduce cumulative impacts (Halpern et al. 2010). In the marine realm, effective PAs can reduce major threats to marine biodiversity loss (e.g., fishing) and deliver significant benefits to habitats and species (Halpern & Warner 2003; Edgar et al. 2014), but shortfalls in management effectiveness and funding often hinder success (Gill et al. 2017). However, if MPAs do not adequately represent species or protect biodiversity from threatening processes, they will be ineffective for near‐term biodiversity conservation regardless of their management effectiveness or the funding they receive. It is unclear whether MPAs are being established where threats they can mitigate (i.e., stoppable threats) occur.

**Figure 1:**
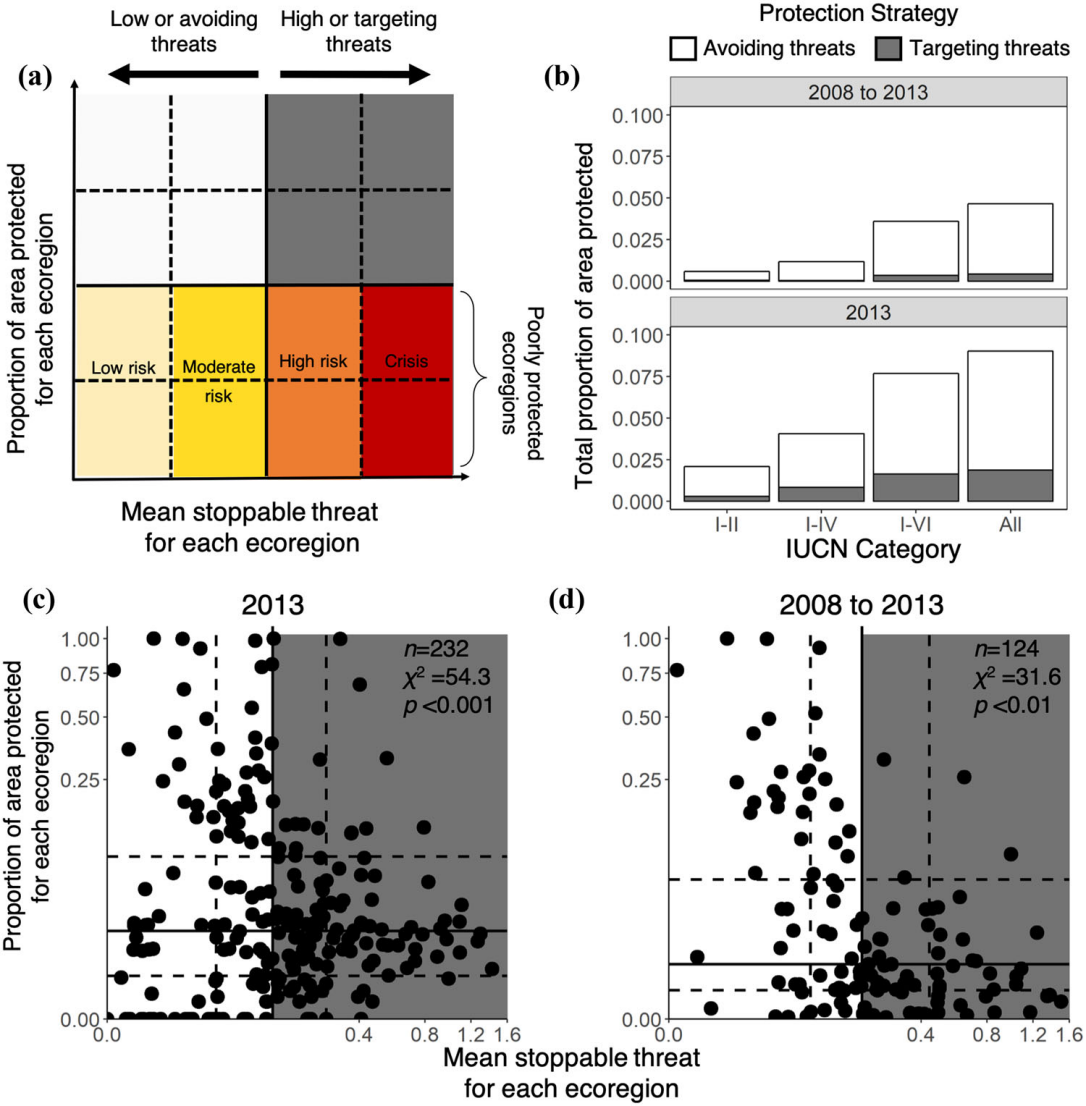
Ecoregion classification scheme and global establishment patterns of marine protected areas in relation to threat: (a) ecoregion classifications by threat and risk categories, (b) proportion of area protected within each protection strategy (avoiding or targeting threat) in 2 periods across International Union for Conservation of Nature protection categories of protection, and the relationship between proportion of area protected and the level of stoppable threat in (c) each global marine ecoregion in 2013 (n = 232 ecoregions) and (d) in ecoregions where protection rose from 2008 to 2013 (n = 124 ecoregions). Axes in (c) and (d) are cube‐root transformed.

We used the most comprehensive data on cumulative global marine threats in 2008 and 2013 (Halpern et al. 2015) to quantify the degree to which MPA establishment has targeted stoppable threats, defined as threats that can be abated through effectively managed MPAs alone. We considered all measures of fishing pressures, benthic structures, and direct human impacts measured by Halpern et al. (2015) in 2008 and 2013 as stoppable (Supporting Information). By combining spatially explicit threat data with information on the location and potential of MPAs to halt threats, we quantified patterns of protection relative to stoppable threats (Fig. 1a) across the world's 232 marine ecoregions (unique biogeographic classifications of global biodiversity patterns for the world's coastal and shelf areas [Spalding et al. 2007]). We developed an impact metric that indicates the comparative amount of protection in high‐ and low‐threat areas within a given MPA estate. We identified 60 high‐risk and crisis areas in need of fine‐scale analyses, where high levels of stoppable threat but very little protection may provide an opportunity for further MPA expansion to reduce threats that compromise biodiversity values. We sought to provide a reference point against which to measure progress in MPA placement in relation to stoppable threats. Such a reference is critical to signatory nations of the Strategic Plan for Biodiversity for development of MPA targets after 2020.

## Methods

We used the 232 marine ecoregions defined by Spalding et al. (2007) to represent global marine biodiversity features. The study was limited to ecoregions and MPAs within EEZs due to challenges of implementing conservation actions beyond national jurisdictions and because the majority of MPA designations and marine threats occur within national waters (Spalding et al. 2007; Halpern et al. 2008). All data sets we used are publicly available. Data on the cumulative human impacts on the world's oceans are available from https://knb.ecoinformatics.org/#view/https://doi.org/10.5063/F19Z92TW, protected area data are available from https://www.protectedplanet.net/, and ecoregion data are available from https://www.worldwildlife.org/publications/marine-ecoregions-of-the-world-a-bioregionalization-of-coastal-and-shelf-areas.

### Marine Protected Areas

The proportion of area protected in each period was estimated by intersecting equal‐area projections of the World Database on Protected Areas (WDPA) (UNEP‐WCMC and IUCN 2017) with marine ecoregions and country EEZs. Terrestrial PAs, those listed as proposed, and UNESCO Biosphere reserves were removed from analysis following WDPA recommendations (UNEP‐WCMC 2016). The MPAs that did not have delineated boundary data were also removed. Missing establishment‐year data were imputed following Butchart et al. (2015) by randomly selecting a year (with replacement) from all PAs within the same country with a known date of establishment. For countries with fewer than 5 PAs with known establishment date, a year was randomly selected from all PAs with a known date of establishment. The random assignment was repeated 1,000 times, and the mean value was assigned to all PAs within each country that were missing establishment dates. Because PAs are managed for different purposes (some allow extractive use), we used reported IUCN classifications as a proxy for management objectives within MPAs. We considered IUCN categories I–II as strict nature reserves, categories I–IV to have been primarily designated for biodiversity conservation, and categories I–VI to include some MPAs that potentially allow some sustainable use of natural resources. The all categories classification included MPAs listed as “not applicable,” “not reported,” or “not assigned” and contained all MPAs within the WDPA database that met our selection criteria. Some countries do not subscribe to IUCN designations and thus meaningfully protected PAs may be excluded by considering only MPAs with IUCN classifications. Furthermore, IUCN classifications refer to only management objectives and not necessarily management effectiveness. Because even ineffective MPAs can provide a basis for more strict and effective management in the future, the results for the all categories classification are presented in the main text.

### Measures of Threat

We considered the impact of 12 threats to marine ecosystems that were measured in 2008 and 2013 (normalized across both periods) from the cumulative human impact data by Halpern et al. (2015). We categorized each threat as stoppable or unstoppable based on the ability of effectively managed MPAs alone to combat each threat (Supporting Information). Stoppable threats have clear marine origins and targets and can therefore be managed through effective marine protection, whereas unstoppable threats often originate from land or diffuse sources that cannot be directly managed through MPA establishment alone. We considered all measures of fishing pressures, benthic structures, and direct human impacts as stoppable. Although shipping threats are stoppable through MPA establishment, shipping was not considered because it was not measured in both periods. Using this information, we calculated the mean level of stoppable threat within each global and country ecoregion with zonal statistics in ArcGIS (version 10.5).

Our framework was built around the goals of MPAs in international conservation agreements and the IUCN definition of PAs (Dudley 2008), which implies that all PAs are first designated for the purposes of conservation. Thus, MPAs should abate any stoppable threat that impedes conservation outcomes. We included benthic structures and direct human impacts in our analysis alongside fishing pressure. Due to potential difficulties in managing or removing existing benthic structures and prohibiting direct human impacts, which largely refer to intertidal trampling and would require strict, zero‐entry protection, we repeated our analysis and considered only fishing pressure as stoppable. Results of this analysis are in Supporting Information.

### MPA Establishment and Stoppable Threats

We assessed the current state of MPA establishment in relation to stoppable threats by comparing the proportion of total area protected with mean stoppable threat in each ecoregion in 2013 and comparing the change in area protected between 2008 and 2013 with mean stoppable threat in 2008. Each ecoregion was classified into 1 of 16 categories based on the quartiles of the proportion of area protected and the level of stoppable threat across all ecoregions (Fig. 1a). We used chi‐square tests to determine whether the observed distribution of protection across ecoregions was independent of threat. If protection was independent of threat (i.e., random), we expected equal numbers of ecoregions (6.25%) in each sector. If MPAs were being established to combat stoppable threats, we expected a greater number of ecoregions with high levels of protection and high levels of threat (top quartile of protection and threat, top left sector Fig. 1a).

To determine the proportion of protection that was targeting or avoiding stoppable threats, we classified ecoregions as low threat or high threat based on the median level of mean stoppable threat across all ecoregions. Low‐threat ecoregions had below the overall median level of mean stoppable threat and high‐threat ecoregions had above the overall median level of stoppable threat (left and right of vertical solid line Fig. 1a, respectively). The MPAs established in low‐threat ecoregions were classified as avoiding threats, whereas those in high‐threat ecoregions were considered to target threats in both periods.

We showcased the general protection strategy globally and in the 20 countries with at least 5 ecoregions in their EEZ and that had protected the greatest proportion of their national waters as of 2013 (Supporting Information). We recalculated the level of protection and the level of stoppable threat within each country's ecoregions. Protection within ecoregions was classified as avoiding threats (protection in low‐threat ecoregions) or targeting threats (protection in high‐threat ecoregions). To increase transparency in reporting, we calculated an impact metric based on the difference between the proportion of protection in high‐threat ecoregions and the proportion of protection in low‐threat ecoregions (see hypothetical example in Supporting Information). Our impact metric was adapted from the conversion‐to‐protection ratio that was developed to calculate global disparities of habitat loss and protection in terrestrial systems (Hoekstra et al. 2005; Watson et al. 2016a) and indicates whether an MPA estate is preferentially targeting or avoiding areas with high stoppable threats. The impact metric (*I*) for country *(C)* is calculated as

(1) $$I_{C}=\;\frac{p_{H}}{a_{H}}-\;\frac{p_{L}}{a_{L}},$$

where *p*_H_ is the area protected in high‐threat ecoregions, *p*_L_ is the area protected in low‐threat ecoregions, *a*_H_ is the area of high‐threat ecoregions, and *a*_L_ is the area in low‐threat ecoregions within each country. The metric is bounded between −1, if only low‐threat ecoregions are protected, and 1, if only high‐threat ecoregions are protected, and results in a value of 0 if high and low‐threat ecoregions are proportionally protected equally. These upper and lower bounds facilitate comparison between countries. The metric itself is useful because it accounts for dependence of the amount of protection in either threat category on the total area of ecoregions within that category. Therefore, a country is not penalized for having less or more area with relatively high levels of stoppable threat. Due to potential costs of establishing MPAs in high‐threat areas and patterns of terrestrial PA trends of avoiding areas of commercial value (Venter et al. 2017), we hypothesized that the majority of leaders in marine protection would be protecting low‐threat ecoregions more than high‐threat ecoregions and would thus have a negative impact metric.

We assessed the performance of MPAs globally and within the top 20 countries in marine protection by comparing the impact metric calculated from the 2013 MPA system with a random solution. For the random‐protection scenario, we selected ecoregions at random (with replacement) and allocated area equal to the median PA size within that country until the total area protected in 2013 was reached (Supporting Information). The median value was used because it is more robust to outliers. We ensured the proportion of area protected in each ecoregion never exceeded 1 and calculated the impact metric as above. This was repeated 1,000 times. The random impact metric was equal to the average impact metric across all simulations. Our null hypothesis was that countries protect area randomly (i.e., regardless of threat) and thus observed and random impact metrics would be similar. An observed impact metric that was outside the 95% confidence interval (CI) of the mean random impact metric was considered significantly different than random. If the distribution of the random impact metric across all simulations was not normal, a bootstrap confidence interval was generated using the boot package in R (version 3.3.3) (Canty & Ripley 2015).

### Poorly Protected Ecoregions

Ecoregions with below the median proportion of area protected across all ecoregions were identified because they had low levels of protection coupled with varying levels of stoppable threat (Fig. 1a). We divided poorly protected ecoregions into 4 risk categories based on the quartile of stoppable threat: low risk (lowest quartile), moderate risk (second quartile), high risk (third quartile), and crisis (highest quartile). We analyzed how many of these ecoregions cross country borders because this may significantly affect success of protection and management. We assessed whether ecoregions that cross country borders are more likely to be poorly protected using Pearson's chi‐square tests. If threat was independent of whether an ecoregion crossed country borders, we expected the same proportion of poorly protected ecoregions to be transboundary or single country as across all 232 global marine ecoregions. Because we defined risk categories of poorly protected ecoregions by quartiles of stoppable threat, we expected 25% of transboundary and 25% of single‐country ecoregions to be in each risk category (Supporting Information).

## Results

### MPA Establishment and Stoppable Threats

**Figure 2:**
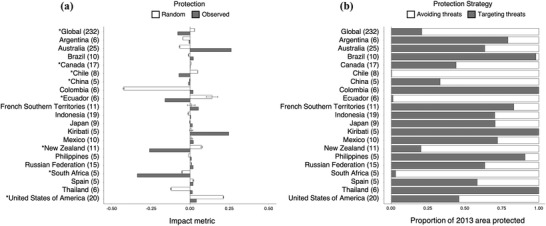
The relative protection of high‐ and low‐threat marine ecoregions globally and in 20 countries: (a) observed and average random (95% CI, n = 1000 random simulations) impact metrics in the 20 countries with the largest marine‐protected‐area estates as of 2013 and (b) the proportion of area protected in 2013 in high‐ and low‐threat ecoregions (parentheses, number of ecoregions in each country; asterisk, random impact metrics significantly worse than random; no asterisk, impact metrics significantly better than random). Impact metric indicates potential of an MPA estate to have a meaningful conservation impact by mitigating stoppable threats and is calculated as the difference between the proportion of high‐threat areas protected and low‐threat areas protected (0, ecoregions of relatively high and low stoppable threat are proportionally protected; negative, ecoregion where relatively low stoppable threats receive greater levels of protection [avoiding stoppable threats]; positive, relatively high stoppable threat ecoregions receive relatively more protection [targeting stoppable threats]).

In 2013, 9.0% of national waters had protection, but only ∼21% were within high‐threat ecoregions (i.e., ecoregions with above the median level of mean stoppable threat, Fig. 1a,b). That is, MPAs covered only 1.9% of ecoregions with high stoppable threat, whereas ecoregions dominated by low levels of stoppable threat had 3.8 times more area protected. These patterns remained consistent across all IUCN categories, but MPAs with the strictest protection (classes I–II) showed the greatest disparity; they had 6.3 times more protection in low‐threat ecoregions than high‐threat ecoregions (Fig. 2b & Supporting Information).

Between 2008 and 2013, nearly 6.3 million km^2^ were protected within MPAs; however, only 9.4% were in ecoregions with high levels of stoppable threat (0.4% of the 4.7% of marine area protected during this period [Fig. 1b]). Within the strictest protection classes (I–II), only 8.4% of protection occurred in high‐threat areas (Fig. 1b & Supporting Information). Protection increased by an average of 14.6% in relatively low‐threat areas and by 1.6% in relatively high‐threat areas. The level of protection within ecoregions was significantly dependent on level of stoppable threat in both periods (*χ* ^2^ = 54.3, *p* < 0.001 in 2013, and *χ* ^2^ = 35.7, *p* < 0.001 2008–2013) (Fig. 1c,d). There were significantly fewer ecoregions with high levels of protection and high stoppable threat than expected from random protection.

Fourteen of the 20 countries with the largest MPA estates (70%) exhibited positive impact indices (Fig. 2a). The majority of these (11) had an impact metric of 0.00–0.05, signifying that low‐threat and high‐threat ecoregions were proportionally protected relatively equally. Australia and Kiribati had the highest impact metrics, 0.26 and 0.25, respectively (Supporting Information contains metrics by country and IUCN category). Chile, Ecuador, and South Africa targeted areas with few stoppable threats almost exclusively (Fig. 2b).

The observed global MPA impact metric performed significantly worse than a randomly placed MPA system (Fig. 2a & Supporting Information). Random MPA allocation resulted in an average of >2.4 times more protection in high‐threat ecoregions than was observed. At the national scale, the United States, New Zealand, Ecuador, South Africa, Chile, Canada, and China had impact metrics that were significantly worse than random, whereas the remaining 13 countries had impact metrics significantly better than random (Fig. 2a & Supporting Information).

### Poorly Protected Ecoregions

**Figure 3:**
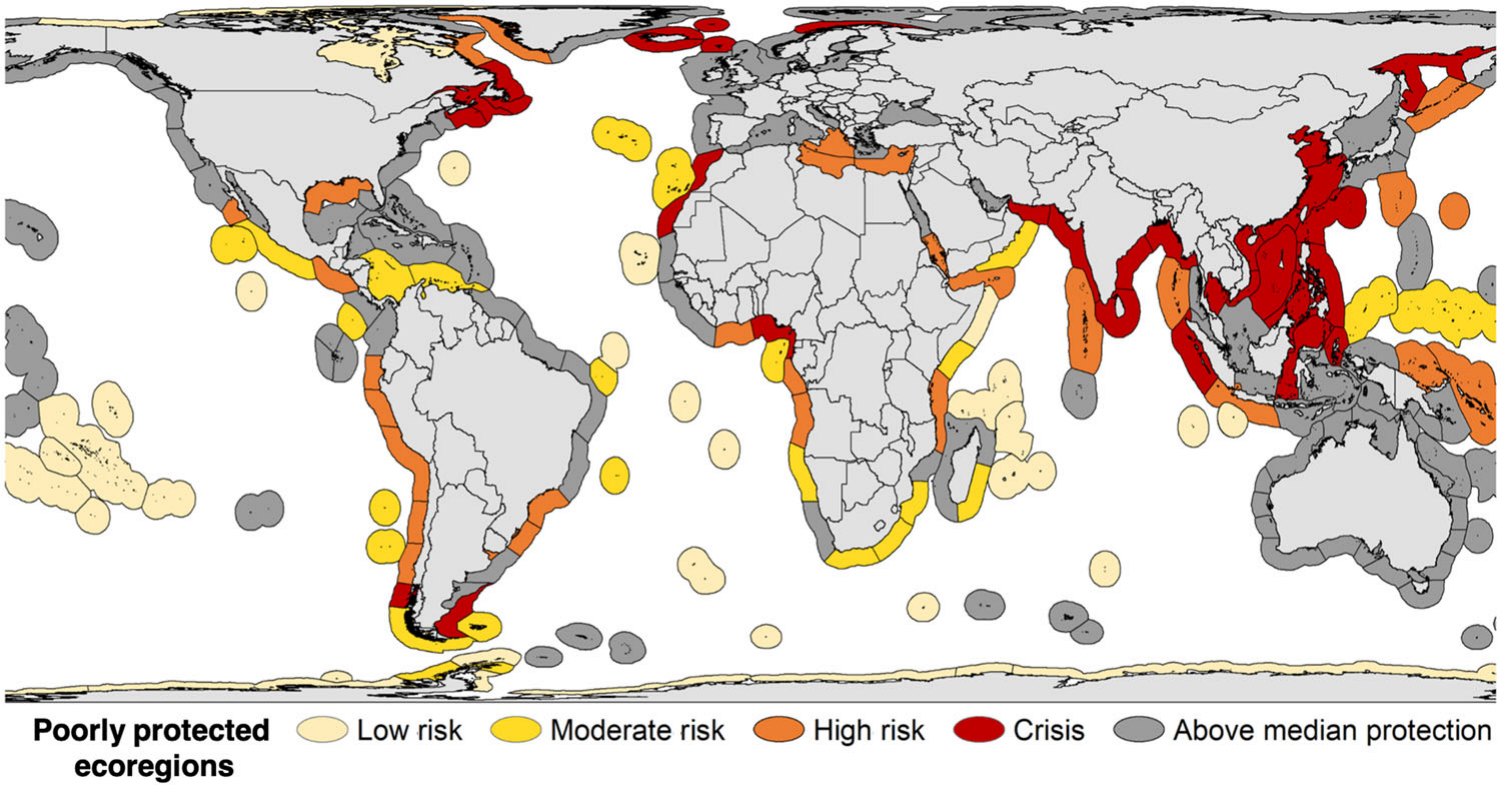
Spatial distribution of poorly protected marine ecoregions across 4 categories of risk from stoppable threats. Ecoregions below median proportion of area protected are divided by quartiles of stoppable threat into 4 risk categories (Fig. 1a): low risk (bottom quartile), moderate risk (second quartile), high risk (third quartile), and crisis (top quartile).

Poorly protected ecoregions (i.e., ecoregions with below median proportion of protection across all ecoregions) occurred across 134 countries. We identified 33 low‐risk (lowest quartile), 23 moderate‐risk, 29 high‐risk, and 31 crisis (highest quartile) ecoregions (Fig. 3). Crisis ecoregions spanned 47 nations but were predominately in the Indo‐Malay region. Of all 232 ecoregions, 85 (36.6%) occurred in a single country and 147 (63.4%) crossed country borders. The risk level of poorly protected ecoregions was significantly dependent on whether an ecoregion crossed country borders. Specifically, significantly more transboundary ecoregions were identified as crisis ecoregions, whereas significantly more low‐risk ecoregions occurred in a single country than expected by chance (*χ* ^2^ = 16.3, *p* < 0.001) (Supporting Information).

## Discussion

Timely, focused action is urgently needed to safeguard marine biodiversity against rapidly increasing threats (Halpern et al. 2015). We found that MPA establishment, a primary component of many conservation efforts, largely avoided abatable threats to biodiversity, and relatively high‐threat ecoregions were significantly less protected than expected by chance. This disparity can overestimate the net short‐term benefits (i.e., conservation progress) MPAs deliver. As countries continue expanding their MPAs to meet current and future international conservation agreements, such as Aichi Target 11, it is crucial to encourage dialogue and consider the relationship between protection and threat to ensure conservation outcomes greater than the counterfactual (i.e., what would have happened in the absence of protection [Ferraro & Pattanayak 2006]).

We identified 31 crisis and 29 high‐risk marine ecoregions where protection was very low but stoppable threats were high. Although poorly protected ecoregions spanned 134 countries, the large concentration of crisis ecoregions in the Indo‐Malaysian region, a global hotspot for marine biodiversity (Roberts 2002), presents broad priorities for MPA establishment to reduce stoppable threats, compensate for protection biases of the current MPA system, and improve overall ocean condition. However, given high political‐capital costs, limited conservation funds, and heavy dependence on fisheries in these areas, this may prove difficult. Conservation incentives such as debt‐for‐nature swaps, an agreement that reduces a country's debt in exchange for a commitment to protect nature, could be critical tools in high‐risk areas. For example, The Nature Conservancy secured the first such swap specifically for marine conservation in the Republic of the Seychelles. Millions of dollars in sovereign debt were paid in exchange for protecting nearly one‐third of its ocean area (Kennedy 2018; Williams 2018). Rising debt in countries like Indonesia, whose debt has increased by nearly 50% in the past 5 years (Soesmanto & Tjoe 2018), could create political momentum for such initiatives. Threat can also be reduced through means other than formal protection, such as programs to promote alternative livelihoods (Ferrol‐Schulte et al. 2013) and strategic international trade mechanisms that enhance domestic food supply and security while potentially decreasing overexploitation of fisheries resources (Kent 1997).

Given the large, ecoregion scale of our analysis, fine‐scale analyses that consider important economic, environmental, and social factors (i.e., livelihoods and resource needs of local people) will be imperative to maximize the conservation impact of future MPA expansion through local‐scale implementation. For example, areas with high threat may also be more costly to protect in terms of opportunity costs (Ban & Klein 2009), management costs, etc. One solution could be to identify and protect the most ecologically intact sites within high‐threat ecoregions (e.g., those with likely higher biodiversity value given their condition) because this is likely to be cheaper to implement, have fewer negative social impacts, and have a higher chance of supporting ecological processes necessary to maintain ecosystem function (Martin & Watson 2016). The size of MPAs that are politically feasible in high‐threat areas may be much smaller than the large, remote MPAs that have been attractive to national governments for quickly reaching area‐based goals with minimal impact on resource users (Singleton & Roberts 2014; Jones & De Santo 2016). However, numerous and relatively small MPAs in high‐threat areas could still contribute to achieving conservation goals, such as representation (Kuempel et al. 2016) and increased larval export and adult spillover to unprotected sites (Carr et al. 2017), which would reduce displacement of threats and allow flexibility to account for the needs of local people (Jones & De Santo 2016) at a finer scale.

Ecoregions that crossed national borders were significantly more likely to be crisis ecoregions. This signifies that human generated political boundaries lead to reduced levels of protection and higher levels of threat, potentially due to questions of responsibility and ownership of the sea (Mackelworth 2012). Given the interconnected nature of marine environments, which do not prescribe to these borders, transboundary cooperation is needed to achieve overall conservation outcomes (e.g., the Coral Triangle Initiative, http://www.coraltriangleinitiative.org). Transboundary initiatives are becoming more prevalent in the global PA estate, but can be complex and challenging to implement (Westing 1998), particularly given conflicts in management strategies and objectives.

Ensuring that both costs and benefits of transboundary conservation and management schemes are fair and equitable among stakeholders has been identified as a key component of ensuring timely and effective conservation outcomes (Campbell & Hanich 2015). Game‐theoretic and review approaches of transboundary fisheries management (e.g., oceanic tuna stocks in the Western and Central Pacific Ocean) suggest that side payments may help resolve this complex problem (Munro 1979; Bhat & Huffaker 2007; Campbell & Hanich 2015) and could be similarly applied in the context of MPAs, but examples of how such burdens should be shared are lacking and context‐dependent, innovative solutions are needed.

Our impact metric is easily calculated and reveals patterns in the proportion of protection in high and low‐threat areas. Although most countries in our analysis protect high and low‐threat areas relatively equally, currently little guidance exists on what this balance should look like to achieve desired outcomes. In 2001, a report by the National Research Council was one of the first to propose that MPA priorities should include vulnerable areas rather than solely achieving area‐based measures, but today, conservation policies offer no consideration of threat, or lack thereof, in reaching PA and conservation goals. Debate about whether to protect the most threatened areas (e.g., Ferraro & Pattanayak 2006; Pressey & Bottrill 2008; Devillers et al. 2015) or the last of the remaining large, intact land and seascapes (i.e. wilderness) (Graham & Clanahan 2013; Watson et al. 2016b, 2018) remains highly contentious, which may affect the generation of clear recommendations.

Because of the rapid deterioration of many habitats from both stoppable and unstoppable threats, large, functioning (in an ecological and evolutionary sense) areas, almost by definition, are irreplaceable (Watson et al. 2018). However, some of these places have relatively low levels of stoppable threat, so they are less likely to benefit from MPA establishment in the short term. Conversely, many habitats, including some that are still large and intact, are highly vulnerable to future threat (such as fishing) and will be heavily affected by threats that MPAs can mitigate. In these areas biodiversity features may be permanently lost if no action is taken (Pressey & Bottrill 2008). Protecting relatively intact habitats in currently low‐threat ecoregions will provide conservation benefits against future threats as threats intensify and cover larger areas of the globe. However, the time scales on which these conservation benefits will be realized may be longer than approaches that deal with direct threatening process in the immediate time horizon.

It is becoming clear to many that a combination of both conserving threatened areas and preserving vulnerable intact habitats is crucial to preserving the full range of biodiversity (i.e., representation) in perpetuity (Watson & Venter 2017; Jones et al. 2018a; Watson et al. 2018). Furthermore, reactive and proactive conservation approaches are likely to attract different funding opportunities, unlocking conservation investment that would have otherwise been unavailable. Determining where and when it is most cost‐effective to establish protection that combats current versus anticipated future threats (Sacre et al. 2019) and whether utilizing conservation resources for one approach precludes the other are high priorities for future research.

Most of the measured stoppable threats in our analysis (Supporting Information) can be attributed to fishing pressure, which is unsurprising considering fishing is one of the most pervasive immediate threats to marine biodiversity (Halpern et al. 2010; Maxwell et al. 2016). When only fishing‐related threats are considered abatable through MPA establishment, the results are quantitatively and qualitatively similar (Supporting Information). Because MPAs largely avoid areas of high fishing pressure, yet are often used as a key fisheries management tool (Worm et al. 2009), their impact in reducing the overexploitation of marine fisheries should be further investigated. Nevertheless, our research shows that the large biases in the location of MPAs in relation to the location of marine threats that MPAs are able to abate needs to be considered in future MPA establishment and conservation targets.

International agreements could help correct the current biases between protection and threat by setting representation goals (e.g., via the process of identifying key biodiversity areas being led by the IUCN [2016]) that span a range of within‐feature threat classes and simultaneously improve conservation metrics to capture both gains and losses for biodiversity (i.e., conservation impact). The former may be particularly important because of the limited understanding of within‐feature variation of habitats and species (Devillers et al. 2015). The latter, although potentially difficult to develop and standardize, would commend protection in areas at high risk of degradation. Our impact metric may make the relationship between MPA establishment and threat more transparent, but metrics that include habitat condition at a finer scale (e.g., McDonald‐Madden et al. 2009) are urgently needed to ensure outcomes for biodiversity.

It is clear that the current areal approach to measuring conservation progress masks biases in MPA designation relative to threat, thereby undermining the goal of international conservation agreements to “safeguard ecosystems, species, and genetic diversity” (Convention on Biological Diversity 2010). That so few MPAs are established in high‐threat locations and that many are unlikely to have sufficient funding to abate threats within their borders (Leverington et al. 2010; Gill et al. 2017) shows how little impact the global MPA estate is having on achieving this goal. However, with so little of the ocean currently under protection and ambitious goals on the horizon, there is still tremendous opportunity to correct these shortfalls and construct a global MPA estate with high, near‐term conservation impact. More strategic placement of MPAs and better, more transparent ways of evaluating progress to ensure the world's marine biodiversity persists are urgently needed.

## Supporting information

A sensitivity analysis considering a different threat classification (Appendix S1), a theoretical depiction of the impact metric (Appendix S2), global protection and threat classifications by IUCN category (Appendix S3), the threat classification scheme (Appendix S4), country‐level summary statistics (Appendices S5 and S6), and transboundary country results (Appendix S7) are available online. The authors are solely responsible for the content and functionality of these materials. Queries (other than absence of the material) should be directed to the corresponding author.
