# Supplementary material for: Quantifying biases in marine‐protected‐area placement relative to abatable threats
Source: Conserv Biol. 2019 May 27;33(6):1350–9. doi: 10.1111/cobi.13340 (PMC6899811; doi:10.1111/cobi.13340)
Supplement: Supplementary file 1 — A sensitivity analysis considering a different threat classification (Appendix S1), a theoretical depiction of the impact metric (Appendix S2), global protection and threat classifications by IUCN category (Appendix S3), the threat classification scheme (Appendix S4), country‐level summary statistics (Appendices S5 and S6), and transboundary country results (Appendix S7) are available online. The authors are solely responsible for the content and functionality of these materials. Queries (other than absence of the material) should be directed to the corresponding author. [file COBI-33-1350-s001.docx]

Global marine protected area establishment largely avoids abatable threats to biodiversity

Caitlin D. Kuempel, Kendall R. Jones, James E.M. Watson and Hugh P. Possingham

Supporting materials:

Appendix 1. Supplementary text of the sensitivity analysis considering a different threat classification

Appendix 2. Diagram depicting the calculation of the ‘impact’ metric in a theoretical country. The impact metric is calculated by first designating ecoregions as low-threat or high-threat within a country. High-threat ecoregions have above the median level of stoppable threat across all ecoregions within the country. Low-threat ecoregions have below the median level of stoppable threat across all ecoregions within the country. Then the total area of high-threat ecoregions (dark gray), the total area of low-threat ecoregions (white) and the amount of protection within high (dark gray with lines) and low (white with lines) threat ecoregions is quantified. The metric is then calculated as the proportion of protection within high-threat ecoregions minus the proportion of protection in low-threat ecoregions

Appendix 3 The relationship between the proportion of area protected and the level of stoppable threat (fishing impacts, benthic structures, direct human impacts (first figure) and fishing impacts only (second figure)) in (A) each global marine ecoregion in 2013 (N=232 ecoregions) and (B) in ecoregions that increased protection between 2008 and 2013 (N=124 ecoregions) within each IUCN classification. Vertical lines denote the quartiles of the proportion of stoppable threat and horizontal lines denote the quartiles of proportion of area protected across ecoregions. Axes are cube root transformed.

**Appendix 4.** Classification of threats based on the degree to which marine protected areas can mitigate each threat (additional information on data layers used can be found in Halpern et al. (2008) and Halpern et al. (2015)). All fishing impacts, benthic structures and direct human impacts were considered as ‘stoppable’ within the main text. Results when only fishing pressure is considered as abatable by MPAs are presented in the Supplementary Text.

**Appendix 5.** Country summary statistics for the 20 leading countries in ocean protection that were used in the analyses**.** Countries that had greater than or equal to 5 marine ecoregions and protected the greatest area between 2008 and 2013 and as of 2013 were considered leaders in marine protection globally.

**Appendix 6.** Country level impact metrics by IUCN classification. The impact metric depicts the difference between the proportion of protection in high-threat areas and the proportion of protection in low-threat areas.

**Appendix 7.** The number of transboundary and single country ecoregions that were expected by chance and observed across risk categories. We would expect 73.5 of these ecoregions to be transboundary and 42.5 to be within one country. We would then expect 10.625 ecoregions within one country to be in each risk category and 18.375 transboundary ecoregions within each risk category. We found that the number of transboundary ecoregions that were identified as poorly protected was not significantly different than expected (χ^2^= 1.931, p =0.59), but that the risk level of these ecoregions was significantly dependent on whether an ecoregion crosses country borders (χ^2^= 16.286, p <0.001). Specifically, significantly more ecoregions that cross country borders were identified as ‘crisis’ ecoregions whereas significantly more ‘low risk’ ecoregions occur in a single country than expected by chance.

Appendix 1

To test the sensitivity of our results to different threat classifications, we considered two threat scenarios. The first scenario defined all threats that could potentially be mitigated through MPAs as ‘stoppable’ and included all measures of fishing pressure, benthic structures, and direct human impacts (results presented in main text). The second approach only considered fishing pressure to be stoppable, given potential difficulties in managing or removing existing benthic structures and prohibiting direct human impacts, which largely refer to intertidal trampling and would require strict, zero-entry protection.

When fishing pressure is the only threat that is considered to be abatable through MPA establishment, stoppable threats range from 0 to 0.96 (median = 0.088). This confirms that most measured stoppable threats included in this analysis can be attributed to fishing pressure and results in qualitative and quantitatively similar results. Since five of the seven stoppable threat layers were related to fishing pressure this is unsurprising. Between 2008 and 2013 threats were slightly higher, ranging from 0 to 1.02 (median = 0.096).

When direct human impacts and benthic structures are considered to be ‘stoppable’ along with fishing, stoppable threats ranged from 0 to 1.5 between 2008 and 2013 (median = 0.13). The higher median level of stoppable threat impacts the classification of high and low-threat ecoregions, thus explaining any differences in protection when these threats are excluded.

**MPA establishment and fishing pressure**

A total of 9.0% of national waters had protection in 2013, but only 2.4% was considered to be in high-threat areas (compared to 1.8%). Ecoregions classified as low stoppable threat had 2.8 (compared to 3.8) times more area protected than ecoregions with high stoppable threat. Additionally, 40 (compared to 41) low-threat ecoregions (34.5%) had met the 10% CBD target in comparison to only 15 (compared to 14) high-threat ecoregions (12.9%). Between 2008 and 2013, nearly 6.3 million km^2^ were protected, however only 9.8% (compared to 9.4%) of this protection targeted ecoregions with high levels of threat (still approximately 0.5% of the 4.7% of marine area protected during this period). The average increase in protection between 2008 and 2013 was 14.4% (compared to 14.6%) in relatively low-threat areas, but just 1.7% (compared to 1.6%) in relatively high-threat areas. The level of protection within ecoregions was still significantly dependent on the level of stoppable threat in both time periods (χ^2^= 42.1, p <0.001 in 2013, and χ^2^= 38.8, p =0.017 between 2008 and 2013, Fig. S2A and S2B). Notably, there were significantly fewer ecoregions with high levels of protection and high stoppable threat than would be expected if protection were protection.

Fourteen countries (70%) still exhibited positive impact indices when only fishing pressure was considered. The majority of these (11, compared to 11) had an impact metric between 0 and 0.05 signifying that low-threat and high-threat ecoregions are proportionally protected relatively equally. Kiribati and Australia still had the highest impact indices, however when only fishing pressure is considered Kiribati has a slightly higher value than Australia at 0.24, while Australia’s impact metric is 0.22. Argentina, Chile, China, Ecuador, New Zealand, and South Africa all still had negative impact indices and Chile, Ecuador and South Africa are targeting areas with low stoppable threats almost exclusively (>97% of protection as of 2013 in low-threat ecoregions).

Appendix 2.


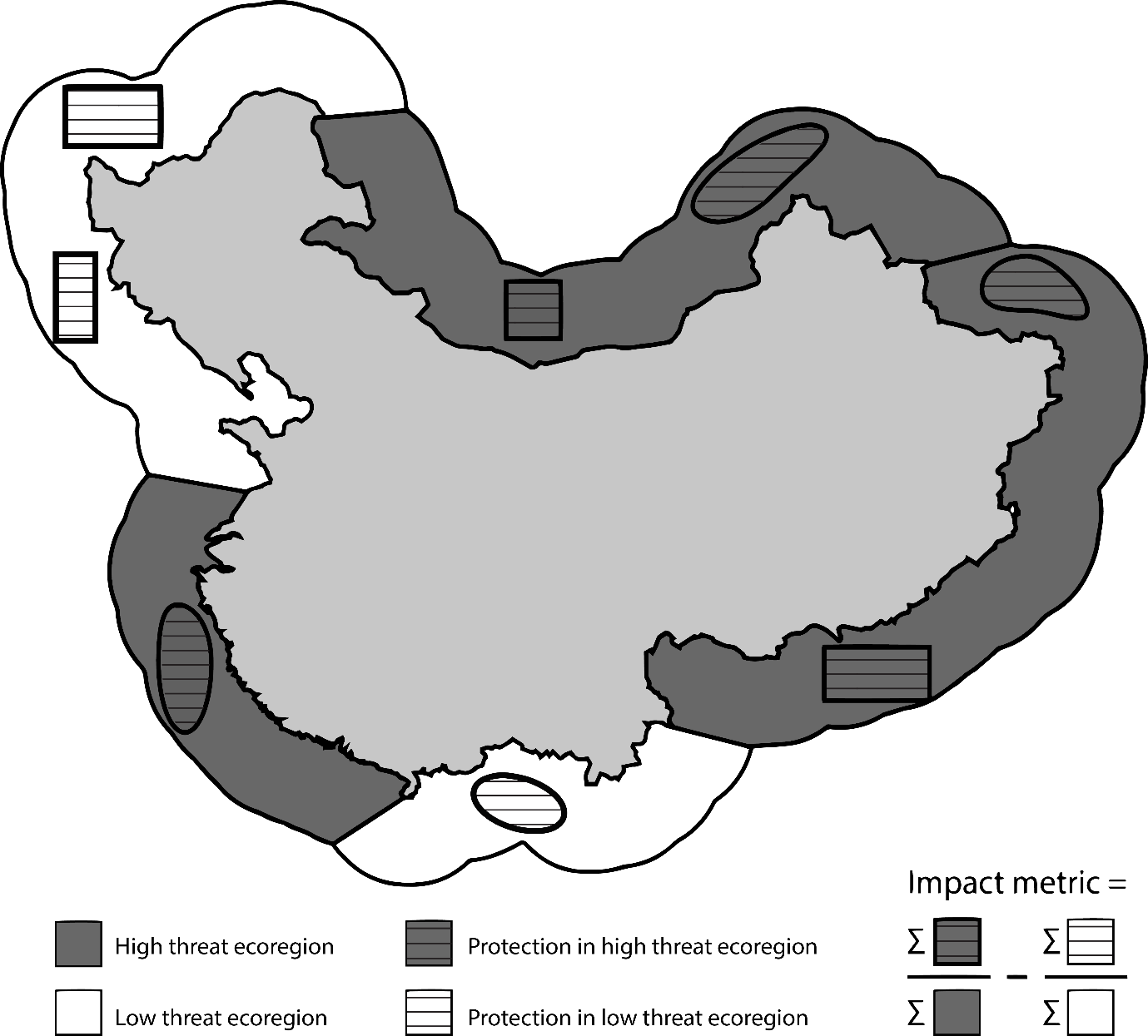


Diagram depicting the calculation of the ‘impact’ metric in a theoretical country. The impact metric is calculated by first designating ecoregions as low-threat or high-threat within a country. High-threat ecoregions have above the median level of stoppable threat across all ecoregions within the country. Low-threat ecoregions have below the median level of stoppable threat across all ecoregions within the country. Then the total area of high-threat ecoregions (dark gray), the total area of low-threat ecoregions (white) and the amount of protection within high (dark gray with lines) and low (white with lines) threat ecoregions is quantified. The metric is then calculated as the proportion of protection within high-threat ecoregions minus the proportion of protection in low-threat ecoregions

Appendix 2.


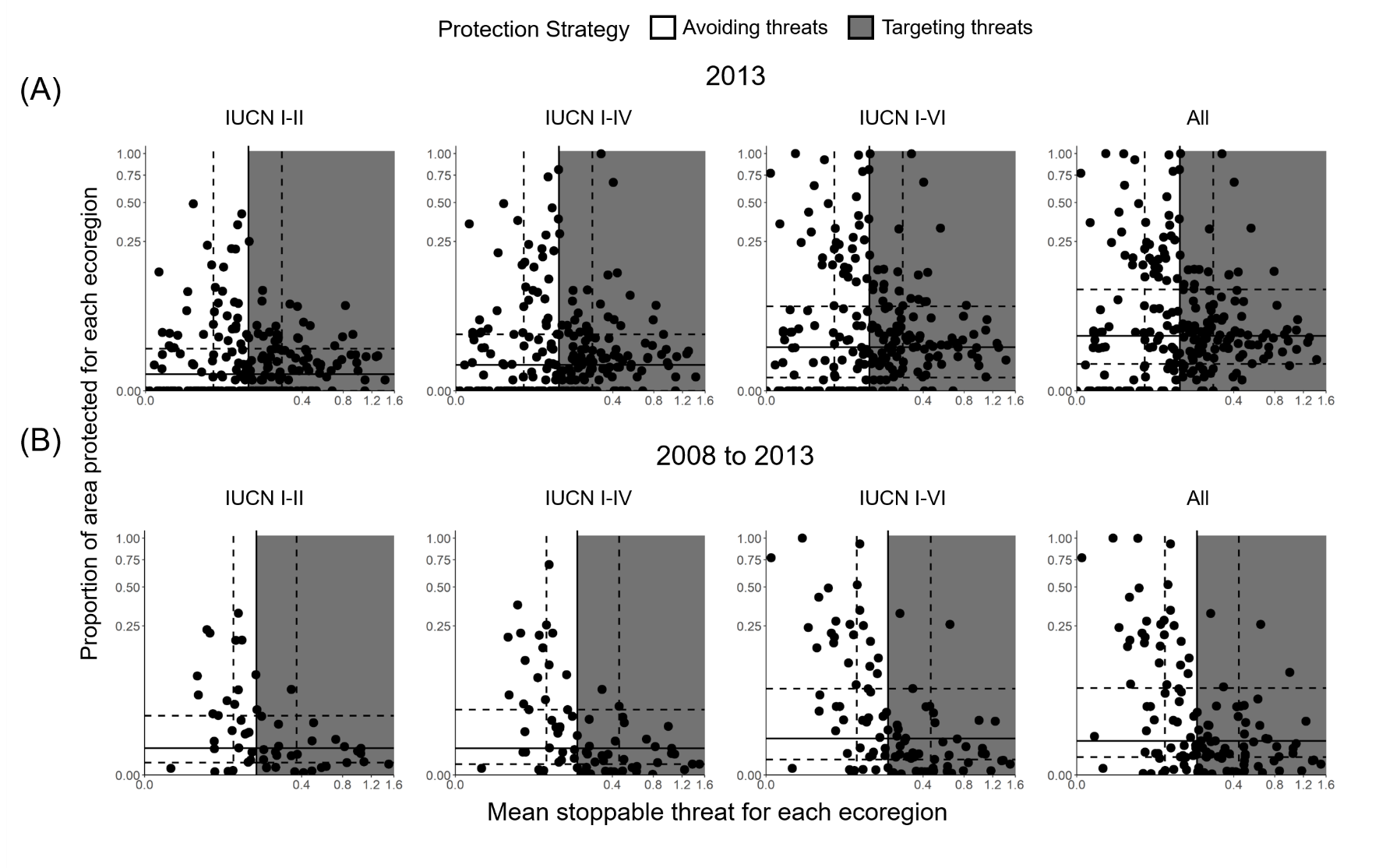


The relationship between the proportion of area protected and the level of stoppable threat (fishing impacts, benthic structures, direct human impacts) in (A) each global marine ecoregion in 2013 (N=232 ecoregions) and (B) in ecoregions that increased protection between 2008 and 2013 (N=124 ecoregions) within each IUCN classification. Vertical lines denote the quartiles of the proportion of stoppable threat and horizontal lines denote the quartiles of proportion of area protected across ecoregions. Axes are cube root transformed.


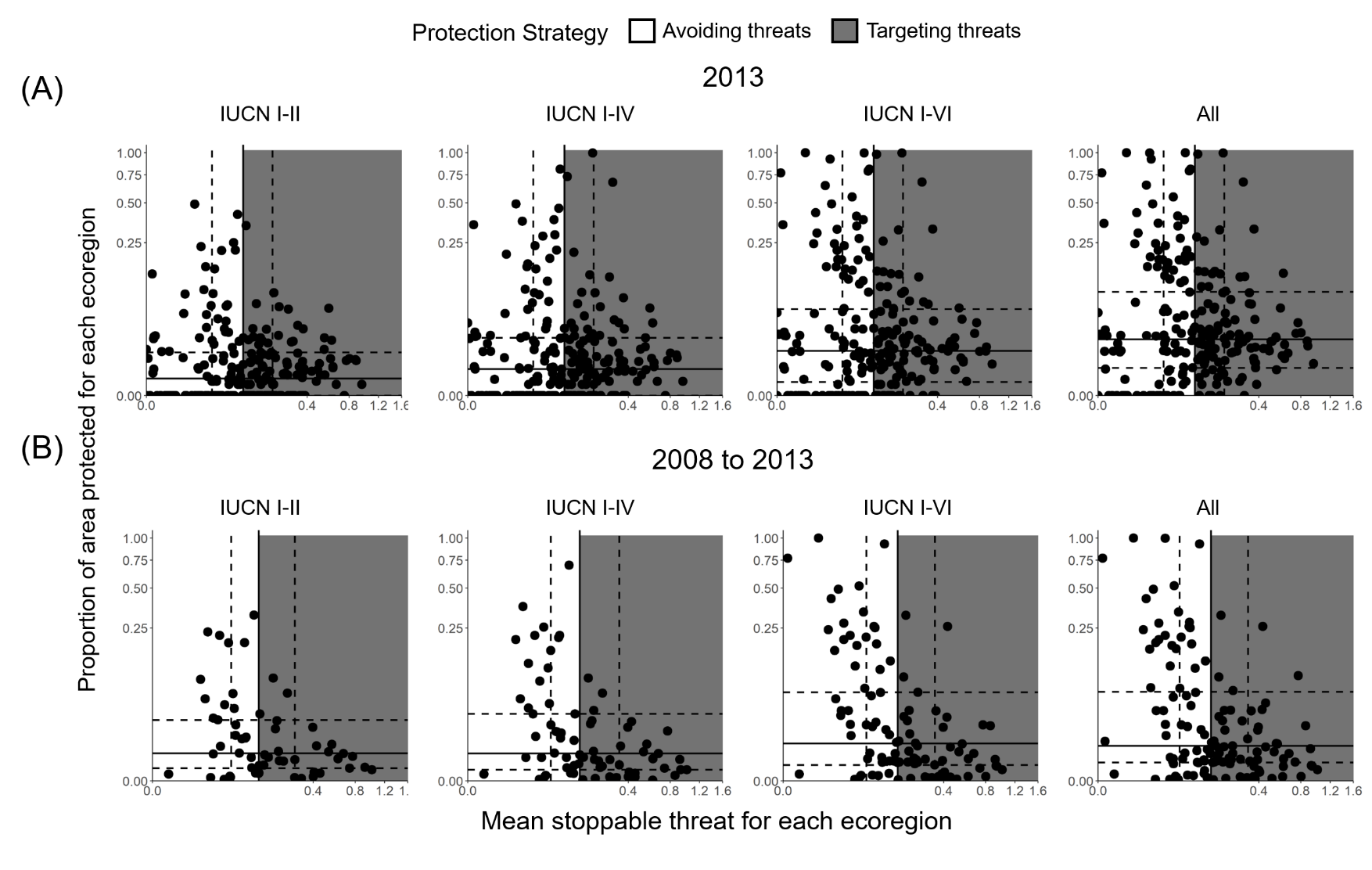


The relationship between the proportion of area protected and the level of stoppable threat (fishing impacts only) in (A) each global marine ecoregion in 2013 (N=232 ecoregions) and (B) in ecoregions that increased protection between 2008 and 2013 (N=124 ecoregions) within each IUCN classification. Vertical lines denote the quartiles of the proportion of stoppable threat and horizontal lines denote the quartiles of proportion of area protected across ecoregions. Axes are cube root transformed.

Appendix 4.

Classification of threats based on the degree to which marine protected areas can mitigate each threat (additional information on data layers used can be found in Halpern et al. (2008) and Halpern et al. (2015)). All fishing impacts, benthic structures and direct human impacts were considered as ‘stoppable’ within the main text. Results when only fishing pressure is considered as abatable by MPAs are presented in the Supplementary Text.

| **Threat** | **Data** | **Threat Category** | **Justification** |
| --- | --- | --- | --- |
| **Fishing** | Demersal destructive fishing | Stoppable | Given effective management, all marine protected areas have the potential to stop fishing pressure within their boundaries. We do note, however, that we focus on the potential for the existing MPA network to reduce fishing pressure, but that many MPAs (e.g. IUCN categories V and VI) are not designated for this explicit purpose. |
|  | Demersal non-destructive fishing (high by-catch) |  |  |
|  | Demersal non-destructive fishing (low by-catch) |  |  |
|  | Pelagic fishing (high by-catch) |  |  |
|  | Pelagic fishing (low by-catch) |  |  |
| **Structures** | Benthic structures (e.g. oil rigs) | Stoppable | Marine protected areas can stop the future development of benthic structures as well as stop or reduce direct human impacts in that area. In this case, benthic structure refers to lights from oil rigs, while direct human impacts uses human population as a proxy for coastal engineering, intertidal trampling, etc. We recognize that existing structures are not likely to be affected and that some MPAs allow developmental activities. |
|  | Direct human impacts |  |  |
| **Pollution** | Organic (pesticide) pollution | Unstoppable | Organic, nutrient, and light pollution originate from land (i.e. land-based drivers). Thus, the establishment of an MPA alone would not directly combat these threats. |
|  | Nutrient (fertilizer) pollution |  |  |
|  | Light pollution |  |  |
| **Climate Change** | Sea Surface Temperature anomalies | Unstoppable | Marine protected areas do not directly address the large-scale drivers of climate change, so will have little effect on reducing threats from sea surface temperature and ultraviolet radiation. |
|  | Ultraviolet Radiation |  |  |

Appendix 5.

Country summary statistics for the 20 leading countries in ocean protection that were used in the analyses**.** Countries that had greater than or equal to 5 marine ecoregions and protected the greatest area between 2008 and 2013 and as of 2013 were considered leaders in marine protection globally.

| **Country** | **Number of ecoregions** | **National area protected in 2013 (km^2^)** | **Proportion of national waters protected in 2013** | **Median protected area size (km^2^)** | **Impact metric** | **Random impact metric** | **Confidence interval** |
| --- | --- | --- | --- | --- | --- | --- | --- |
| Global | 232 | 12138515.0 | 0.09 | 24.06 | -0.0788 | 0.0291 | (0.0286,0.0296) |
| Argentina | 6 | 18229.96 | 0.018 | 10.61 | -0.0070 | -0.0458 | (-0.04587,-0.0457) |
| French Southern Territories | 11 | 61241.27 | 0.036 | 2067.37 | 0.0532 | 0.0074 | (-0.0176,0.0324) |
| Australia | 25 | 2520041 | 0.400 | 9.07 | 0.2619 | -0.0668 | (-0.0696,-0.0639) |
| Brazil | 10 | 46335.96 | 0.014 | 38.94 | 0.0211 | -0.0085 | (-0.0100,-0.0069) |
| Canada | 17 | 44998.68 | 0.009 | 11.62 | 0.0014 | 0.0062 | (0.0056,0.0068) |
| Chile | 8 | 146897.6 | 0.047 | 1.52 | -0.0707 | 0.0487 | (0.0480,0.0494) |
| China | 5 | 5320.062 | 0.006 | 16.02 | -0.0098 | -0.0023 | (-0.0038,-0.0007) |
| Colombia | 6 | 13871.03 | 0.019 | 247.94 | 0.0193 | -0.4231 | (-0.4274,-0.4188) |
| Ecuador | 6 | 140112.9 | 0.131 | 332.06 | -0.1597 | 0.1400 | (0.1040,0.1759) |
| Spain | 5 | 15728.16 | 0.017 | 32.86 | 0.0193 | 0.0206 | (0.0173,0.0239) |
| Indonesia | 19 | 167871 | 0.028 | 50.36 | 0.0052 | -0.0120 | (-0.0141,-0.0099) |
| Japan | 9 | 91249.38 | 0.027 | 40.70 | 0.0149 | -0.0012 | (-0.0034,0.0010) |
| Kiribati | 5 | 412518.5 | 0.125 | 206259.23 | 0.2466 | 0.0080 | (-0.0026,0.0186) |
| Mexico | 10 | 50925.24 | 0.016 | 768.13 | 0.0209 | 0.0095 | (0.0017,0.0173) |
| New Zealand | 11 | 914791.6 | 0.272 | 21.95 | -0.2584 | 0.0742 | (0.0689,0.0796) |
| Philippines | 5 | 20229.69 | 0.011 | 11.80 | 0.0090 | -0.0097 | (-0.0097,-0.0096) |
| Russian Federation | 15 | 210811.4 | 0.030 | 52.67 | 0.0200 | 0.0078 | (0.0059,0.0097) |
| Thailand | 6 | 4376.714 | 0.014 | 119.84 | 0.0150 | -0.1213 | (-0.1229,-0.1198) |
| United States of America | 20 | 3193965 | 0.404 | 6.89 | 0.0405 | 0.2121 | (0.2101,0.2141) |
| South Africa | 5 | 136850.1 | 0.104 | 2.33 | -0.3362 | -0.0512 | (-0.0532,-0.0492) |

Appendix 6.

Country level impact metrics by IUCN classification. The impact metric depicts the difference between the proportion of protection in high-threat areas and the proportion of protection in low-threat areas.

| **Country** | **Impact metric** | | | | |
| --- | --- | --- | --- | --- | --- |
|  | **IUCN I-II** | **IUCN I-IV** | **IUCN I-VI** | **All** |  |
| **Argentina** | 0.0009 | 0.0017 | 0.0103 | 0.0163 |  |
| **French Southern Territories** | 0 | -0.0048 | 0.0505 | 0.0532 |  |
| **Australia** | 0.0892 | 0.1142 | 0.2637 | 0.2613 |  |
| **Brazil** | 0.0015 | 0.0015 | 0.0205 | 0.0211 |  |
| **Canada** | -0.0055 | -0.0054 | -0.0028 | 0.0011 |  |
| **Chile** | -0.0691 | -0.0707 | -0.0707 | -0.0707 |  |
| **China** | 0.0007 | 0.0007 | 0.0003 | -0.0071 |  |
| **Colombia** | 0.0023 | 0.0121 | 0.0121 | 0.0121 |  |
| **Ecuador** | No protection | No protection | No protection | -0.1597 |  |
| **Spain** | 0.0004 | 0.0004 | 0.0177 | 0.0193 |  |
| **Indonesia** | -0.0051 | -0.0045 | 0.0046 | 0.0048 |  |
| **Japan** | -0.0002 | 0.0004 | 0.0117 | 0.0117 |  |
| **Kiribati** | No protection | No protection | 0 | 0.2466 |  |
| **Mexico** | 0.0029 | 0.0055 | 0.0206 | 0.0209 |  |
| **New Zealand** | -0.0006 | -0.0006 | -0.2599 | -0.2584 |  |
| **Philippines** | 0.0013 | 0.0032 | 0.0085 | 0.009 |  |
| **Russian Federation** | -0.0083 | 0.0201 | 0.0178 | 0.0179 |  |
| **Thailand** | 0.015 | 0.015 | 0.015 | 0.015 |  |
| **United States of America** | -0.2876 | -0.0455 | 0.0432 | 0.0405 |  |
| **South Africa** | No protection | No protection | No protection | 0.0019 |  |

Appendix 7.

The number of transboundary and single country ecoregions that were expected by chance and observed across risk categories. We would expect 73.5 of these ecoregions to be transboundary and 42.5 to be within one country. We would then expect 10.625 ecoregions within one country to be in each risk category and 18.375 transboundary ecoregions within each risk category. We found that the number of transboundary ecoregions that were identified as poorly protected was not significantly different than expected (χ^2^= 1.931, p =0.59), but that the risk level of these ecoregions was significantly dependent on whether an ecoregion crosses country borders (χ^2^= 16.286, p <0.001). Specifically, significantly more ecoregions that cross country borders were identified as ‘crisis’ ecoregions whereas significantly more ‘low risk’ ecoregions occur in a single country than expected by chance.

| Ecoregion Type | Expected | Observed | | | |
| --- | --- | --- | --- | --- | --- |
|  | In each risk category | Crisis | High | Moderate | Low |
| Transboundary | 18.375 | 27 | 20 | 15 | 13 |
| Single country | 10.625 | 4 | 9 | 9 | 20 |
